# Influence of Socio-Demographic Factors in Patients With Cutaneous Lupus Erythematosus

**DOI:** 10.3389/fmed.2022.916134

**Published:** 2022-07-11

**Authors:** Amanda M. Walker, Grace Lu, Shari C. Clifton, Motolani E. Ogunsanya, Benjamin F. Chong

**Affiliations:** ^1^Department of Dermatology, University of Texas Southwestern Medical Center, Dallas, TX, United States; ^2^Health Sciences Library and Information Management, The University of Oklahoma Health Sciences Center, Oklahoma City, OK, United States; ^3^Department of Pharmacy, Clinical and Administrative Sciences, The University of Oklahoma Health Sciences Center, Oklahoma City, OK, United States

**Keywords:** cutaneous lupus, autoimmunity, race, socio-demographic factors, health equity

## Abstract

Cutaneous lupus erythematosus (CLE) is a chronic autoimmune skin disease with potential for systemic involvement, disfigurement, and significant disease burden. The relationships of demographics and socioeconomic status with patients with CLE are emerging topics with important clinical implications. The primary objective of our study is to perform a literature review of studies that have investigated demographic and socioeconomic factors amongst patients with CLE and determine whether these factors influence diagnosis frequency, disease severity and outcomes or health related quality of life. We searched multiple databases to identify literature addressing CLE and concepts such as race, ethnicity, gender, income, education level and geographic location. Information regarding primary research objective was extracted from all full text articles, and a summary of findings was prepared. We found that race and ethnicity can influence CLE diagnosis frequency and disease outcomes. Chronic cutaneous lupus (CCLE) occurs more frequently in Black patients, often with higher overall disease damage. Differences between genders exist in CLE in terms of health-related quality of life, as female gender was a risk factor for worse quality of life in several studies. Lower income, low educational attainment, and lack of health insurance all contribute to poorer overall outcomes in CLE patients. This review will help inform physicians about populations at risk for potentially worse outcomes to guide treatment decisions for patients with CLE and provide important information to design interventions that address modifiable social determinants of health in this population.

## Introduction

Cutaneous lupus erythematosus (CLE) is an autoimmune disorder associated with a broad spectrum of cutaneous manifestations. It may progress into systemic lupus erythematosus (SLE) and involve multiple organ systems (1). CLE can be further classified into acute, subacute, or chronic subtypes according to the clinical characteristics of the skin lesions (2). These CLE subtypes vary in clinical presentation amongst different races and ethnicities.

Prior studies have identified racial and ethnic disparities in dermatological diagnosis frequency, clinical presentation, disease management, and patient outcomes (3, 4). With regards to lupus erythematosus, most of the existing literature has focused on the influence of sociodemographic factors in SLE patients, with limited studies examining the same factors in CLE patients (5–7). This underscores the need for more information on factors influencing CLE outcomes to help guide patient care. The primary aim of our study is to perform a literature review examining demographic and socioeconomic factors that can influence CLE diagnosis frequency, disease severity and outcomes or health related quality of life. Detailed search strategy and methods are available in Supplementary Figure 1.

## Race and Ethnicity

Studies investigating racial and ethnic inequities in SLE have suggested higher incidence and prevalence in non-White populations (8). Few studies have investigated the incidence and prevalence of CLE in the absence of SLE, with fewer performed in mixed race populations (9–14). These studies suggest that race and ethnicity do affect CLE diagnosis frequency. Several studies found DLE to occur more frequently in Black or Hispanic patients compared to other races and ethnicities (10, 13, 15, 16). A cross-sectional study from the Georgia Lupus Registry showed that the highest age-adjusted incidence rates for DLE were seen in Black women (6.6 for definite DLE and 7.9 per 100,000 person years for definite and probable DLE) (10). These results are also echoed in the Manhattan Lupus Registry, which showed a prevalence of primary DLE that is significantly higher among non-Latino Blacks (23.5 per 100,000 person-years) and Latinos (8.2), and DLE incidence higher among non-Latino Blacks (2.4) (13). A large, multi-ethnic SLE longitudinal cohort study investigating the occurrence of discoid rash found that DLE was more common in Blacks (*p* < 0.001) (15). Black and Hispanic patients were not the only populations shown to be at higher risk of CLE. A study from New Zealand reported that the Maori/Pacific portion of their population had a greater relative risk of all types of CLE combined vs. their European counterparts (2.47, 95% CI 1.67–3.67) and a higher relative risk of DLE overall (5.96, 95% CI 3.06–11.6) (14).

While types of CCLE have been shown to occur more frequently in non-White populations, the opposite is true for SCLE (14, 16). A study examining skin damage and impact on quality of life in CLE patients found that 57 (97%) of all cases of SCLE occurred in White patients and no cases of SCLE occurred in Black patients (16). Jarrett et al. reported that although not statistically significant, the Maori/Pacific population in New Zealand has an overall decreased relative risk of SCLE compared to European participants (14). These differences in subtype distribution also have implications for disease course, as patients with SCLE can expect significant improvements in their skin disease activity over a shorter period of time than those with chronic cutaneous lupus (CCLE) (17).

Racial and ethnic differences can impact CLE disease course and severity. A study from Australia found that non-indigenous patients presented to clinic sooner than indigenous patients (18). Verma et al. found that Black patients develop CLE at an earlier age than non-Black patients and present with greater initial disease damage (16). Not only are non-White patients younger at initial disease presentation but Black patients may be more likely to hospitalized longer than their White counterparts (14.5 days vs. 6.3 days, respectively, *p* < 0.02) (19). When comparing DLE lesion distribution and disease activity and damage scores using the Cutaneous Lupus Erythematosus Disease Area and Severity Index (CLASI) between Black vs. non-Black patients, Black patients with DLE (median = 10.0, IQR 6.0–14.5) had higher baseline CLASI damage scores compared with non-Black patients (median = 6.0, IQR 3.0–10.0) (*p* < 0.001) (20). Black patients were also more affected by the presence of dyspigmentation in any location compared to non-Black patients (99 vs. 79%, *p* < 0.001). In addition to higher baseline CLASI damage scores, Blacks were less likely to see ≥40% improvement in CLASI damage scores compared to other races (17). Interestingly, Ker et al. found that there was significant improvement in CLASI activity scores over time in patients who were Black (*p* = 0.049), Hispanic or Asian (*p* = 0.02) (21). Additional factors may contribute to disease presentation differences such as disparities in access to health care and insurance status. Non-White patients represent the largest proportion of uninsured patients with 16.7% of Hispanic and 9.6% of Black patients reporting no insurance, while White patients had the highest rate of private insurance at 75.2% (3). Lack of insurance can lead to delays in treatment in non-White patients which may contribute to greater severity of disease at time of presentation. In addition, the burden of disease in uninsured patients is likely higher, which may also influence disease outcomes (22).

Race and ethnic differences were also noted in co-existing medical conditions in CLE patients. A cross-sectional study of CLE patients looking for risk factors for coexistent autoimmune disease in CLE found that CLE patients with at least one coexisting autoimmune condition(s) were more likely to be White (odds ratio [OR], 2.88; 95% CI, 1.00–8.29; *p* = 0.0498) (23). Black patients also have significantly lower 25-OH vitamin D levels compared to Caucasian and Hispanic patients when controlling for CLE disease status, leaving them more vulnerable to vitamin D insufficiency when practicing photoprotective behaviors that often coincide with the CLE diagnosis (24).

Increased disease severity in CLE has been shown to be associated with worse quality of life (25). However, when looking for differences in quality of life by race or ethnicity, neither Klein et al. or Vasquez et al. found any significant associations (7, 25). In contrast, Verma et al., did find that CLASI damage scores in Black patients significantly correlated with SKINDEX-29+3 symptom scores but not in White patients (16). Similarly, a study investigating depression in a primarily Black population in Georgia found that 26% of Black CLE patients reported moderate to severe depression. Higher or worse SKINDEX-29+3 symptoms, functioning, and CLE-specific domain scores were significantly associated with depression as well (26).

## Gender

CLE incidence and prevalence rates are higher in female patients (9, 10, 12–14). These studies report female to male gender ratios ranging from 1.79:1 to as high as 9:1 (9, 12). The exception to this finding comes from a predominately White population study in Minnesota which found the prevalence of CLE to be higher than SLE in men than in women (11). All studies investigating alterations in disease course or severity were predominately female cohorts (16–21, 25). Gender did not play a significant role in predicting progression of disease activity in a multi-center endeavor studying the natural disease course of CLE (21). Similarly, no other studies found significant differences in natural CLE disease course or severity between males and females (16–20, 25).

Studies investigating health-related quality of life and depression in CLE patients report that depression is increased in CLE patients compared to the non-CLE population (27). Most studies in CLE patients found female gender to be a risk factor for poor quality of life or depression (7, 25, 28–30). Klein et al. found that female gender was associated with poor quality of life in all SKINDEX 29+3 subdomains (*p* < 0.006) (25). Results from a multi-center study comparing two different cohorts of CLE patients also found that being female was significantly associated with poor quality of life in all SKINDEX-29+3 subdomains and in SF-36 physical functioning, role-physical, and bodily pain scores (7). A small Japanese cohort found female gender to be a risk factor for poorer quality of life and that functioning and emotions subscales were greater in females compared to males (29). Similarly, Teske et al. found that female DLE patients were significantly associated with poorer QoL in the SKINDEX-29+3 emotions domain, lupus specific scores and symptoms scores (30). Possible reasons as to why females are more susceptible to poorer quality of life may be related to other social factors and increased psychiatric comorbidities related to cosmetic appearance as seen in other skin diseases like acne and vitiligo (31, 32). CCLE can causes significant scarring and hair loss, which may affect women's self-esteem more than men and promote seclusion.

Although most studies show that females are more susceptible than males when it comes to health-related quality of life, two studies found no statistical difference between males and females. This could be related to small sample size of males in each cohort, making robust comparisons difficult. Hesselvig et al. actually found that men with CLE had a higher risk of depression than women (27). However, a small cohort of LE patients in Madagascar did not find any significant correlations between gender and Dermatology Life Quality Index (DLQI) scales (33). Similarly, a CCLE cross-sectional cohort study in Georgia did not find significant differences in risk and severity of depression by gender (26). It is possible that males may be more affected by lack of social support compared to females. A comparison of quality of life in a cohort of SLE patients found that male patients scored lowest in social support domains, and was attributed to differences in communication styles between males and females (34).

## Income, Education, and Geographic Location

Socio-demographic factors including income, education level, and geographic location can affect patients with CLE (7, 19, 28, 30, 33, 35). CLE patients have been shown to experience significantly high healthcare expenditures, particularly if depression is present. In CLE patients with depression, inpatient visits (*p* < 0.01), prescription drugs (*p* < 0.001), and ER visits (*p* < 0.05) were identified as the most significant contributors to medical expenses, respectively (28). Higher levels of poverty were associated with a significantly increased likelihood of rehospitalization within 1 year (*p* < 0.0047) and being female (*p* < 0.002) (19). Income level was also shown to have an inverse relationship with CLASI damage scores (*p* = 0.006), but no significant relationship to CLASI activity scores (35).

In contrast, existing literature has identified variable associations between income and health-related quality of life (7, 30, 33, 35). An annual income < $10,000 was associated with a lower quality of life in the SKINDEX-29+3 functioning and symptom domains in three studies (7, 30, 35). Annual income < $10,000 corresponded to poorer SKINDEX-29+3 symptoms (*p* = 0.002) and lupus-specific (*p* = 0.02) sub-domain scores (7). Joseph et al. also found significant differences in SKINDEX 29+3 lupus-specific (*p* < 0.05), functions, symptoms (*p* < 0.01), and emotions subdomains (*p* < 0.001) between the < $10,000 and >$50,000 annual income categories (35). Within this cohort, Whites comprised the largest proportion in the highest income bracket (58.5%), whereas Blacks comprised a majority of the lowest income bracket (67.1%) (*p* < 0.001). Similarly, when looking at a primarily Black cohort with CCLE, Hong et al. found employment was associated with a lower likelihood of depression (OR = 0.24, *p* < 0.01). Although annual visits with a dermatologist or rheumatologist was not statistically significant, they also found that insured patients and those who visited a PCP in the last year were at lower risk of depression (26). However, Sendrasoa et al. identified an inverse relationship between income and health-related quality of life, with high monthly income associated with increased impairment of quality of life (33). This difference may due to those with higher monthly income having different healthcare expectations than those with low monthly income in Madagascar.

Few studies examined educational attainment (15, 23, 33). Interestingly, CLE patients with at least one coexisting autoimmune condition were less likely to have high school level of education (23). Other studies have also found differences in disease presentation or quality of life depending on degree of educational attainment. In a cohort of SLE patients with CLE, those with DLE were more likely to have fewer years of formal education (15). In addition, patients with a low-level of education had poorer DLQI scores than those with a mid or high education level (*p* = 0.008) (33).

It is well-known that increased sun exposure and ultraviolet (UV) radiation are associated with increased skin flares in those with CLE. Thus, differences in geographic location may have some influence on diagnosis frequency or disease outcomes in CLE. Only one study has directly compared CLE outcomes between two different locations in the United States (7). Investigators found that although most quality of life measures were very similar between the two CLE populations, there were significant differences in Skindex-29+3 functioning and lupus-specific subdomains and SF-36 physical functioning, role-physical and general health subscales between CLE patients seen in Dallas, Texas, vs. Philadelphia, Pennsylvania (7). CLE patients from the Dallas metroplex expressed an increased tendency to stay inside and were more likely to avoid outdoor activities for fear of increased UV exposure. Despite growing evidence for the effects of UV radiation on CLE lesions, it does not appear that location, and therefore the amount of solar radiation is associated with worse CLE outcomes. Scolnik et al. analyzed cutaneous findings, including DLE, in SLE patients according to latitude and degree of solar radiation and found that living in a city with higher daily solar radiation was not associated with increased cutaneous manifestations during follow up (36).

## Discussion

**Figure 1:**
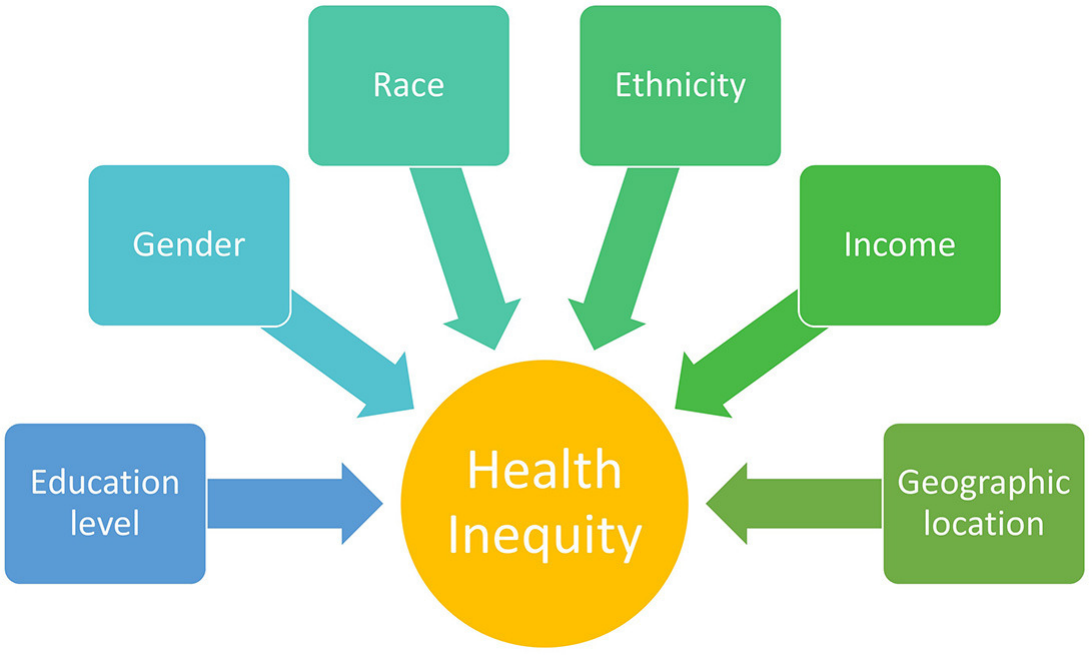
Sociodemographic factors contributing to health inequity that have been investigated in patients with cutaneous lupus erythematosus (CLE).

We present a summary of current research findings related to socio-demographic factors in patients with CLE (Figure 1). There is strong evidence that CCLE, in particular DLE, more commonly affects non-White patients (10, 13, 15, 16). Physicians are encouraged to be aware of these differences when counseling their non-White patients regarding expected disease course and manage expectations for treatment outcomes. Furthermore, their communication styles with patients about their skin disease may affect their quality of life. Depression in Black patients with CCLE was directly associated with worse reports of staff disrespect and inversely associated in patients who reported that their physicians explained their labs and medications (26).

Differences in disease severity in non-White patients might be partially explained on a genetic level. Recent whole blood transcriptome data identified six patient subsets with distinct molecular phenotypes separated by race and CLE subtype, with increased T-Cell signatures in subsets predominated by Blacks and DLE subtype (37). Further studies are needed with greater CLE subtype diversity and larger sample sizes to investigate the influence of these molecular differences which may help to improve treatment options and outcomes in these groups by developing tailored treatment plans based on molecular subtype.

Like most autoimmune diseases, CLE occurs primarily in female patients; however, it does not appear that female gender is associated with worse CLE disease course and severity. Female patients may differ from males in their attitudes toward their appearance and place emphasis on cosmetic appearance. Further studies with larger sample sizes of male participants are needed in order to accurately compare disease severity and health-related quality of life between these two groups. We recommend that physicians be open to screening for comorbid psychiatric conditions like depression or suicidal ideation so that they may refer them to the appropriate services or online support groups.

Finally, socioeconomic factors like reduced income and low educational attainment have been associated with worse outcomes in patients with CLE (7, 15, 19, 23, 26, 30, 33, 35). A diagnosis of CLE comes with significant financial burden. Inadequate funds or insurance coverage may lead to some patients not being able to afford their treatments, contributing to increased CLE damage and poorer overall outcomes (35). Those with lower socioeconomic status may be more susceptible to social isolation or increased sense of discrimination, contributing to poorer quality of life (35). Furthermore, low educational attainment has been associated with low socioeconomic status which may contribute to low health literacy levels (38). Low health literacy negatively impacts health-related outcomes, use of preventive services, and a patient's overall ability to access care. Possible interventions for improving CLE outcomes in these patients may include increasing physician reimbursement for Medicaid patients, instituting educational videos and patient lectures to improve health literacy, and increasing non-White participation in clinical trials and research to increase representation of these populations.

In summary, differences in CLE diagnosis frequency, disease outcomes, health related quality of life exist depending on the socio-demographic factors present. We have identified that race and ethnicity play a large role in CLE diagnosis frequency and disease outcomes. Although racial and socio-demographic inequities appear to exist in CLE, further investigation is necessary to improve care, increase awareness, and further develop interventions aimed at tackling health inequity in CLE.

## Author Contributions

AW, MO, and BC contributed to conception and design of the study. AW, GL, and SC contributed to the acquisition and analysis of the data. AW and GL drafted the manuscript. All authors contributed to manuscript revision, read, and approved the submitted version.

## Funding

AW was supported by the Rheumatology Research Foundation Medical Student Preceptorship award under Marc R. Chevrier, MD, PhD, FACR, Lupus Research Memorial Fund.

## Conflict of Interest

BC is an investigator for Daavlin Corporation and Biogen Incorporated and Pfizer Incorporated. He is a consultant for Bristol Meyers Squibb, EMD Serono, Horizon Therapeutics, and Biogen Incorporated. He receives royalties from MAPI Research Trust. The remaining authors declare that the research was conducted in the absence of any commercial or financial relationships that could be construed as a potential conflict of interest.

## Publisher's Note

All claims expressed in this article are solely those of the authors and do not necessarily represent those of their affiliated organizations, or those of the publisher, the editors and the reviewers. Any product that may be evaluated in this article, or claim that may be made by its manufacturer, is not guaranteed or endorsed by the publisher.

## Supplementary Material

Supplementary Figure 1 Study selection and screening methods used in present study. Figure was adapted from the PRISMA Flow Diagram 2020 V1. Inclusion criteria included studies with cohorts of CLE patients (with or without SLE) whose primary outcome included CLE diagnosis frequency, CLE disease outcomes or health-related quality of life. Exclusion criteria included non- English studies, conference abstracts, and SLE only cohorts. Searches were completed on February 2, 2022. There were no publication date limits. Covidence review platform was used for study screening and extraction. Two separate reviewers (AW and GL) independently appraised all studies meeting inclusion and exclusion criteria. 1,326 studies were imported to Covidence for abstract and title screening. 302 duplicates were removed and then 1,024 studies were left for screening. 982 articles were excluded based on exclusion criteria. 42 articles were assessed for full-text eligibility and 17 studies were excluded, leaving 25 studies total included in this review. Information regarding primary research objective was then extracted from all full text articles and summarized in text.
