# Supplementary figures and images for: Influence of Socio-Demographic Factors in Patients With Cutaneous Lupus Erythematosus
Source: Front Med (Lausanne). 2022 Jul 11;9:916134. doi: 10.3389/fmed.2022.916134 (PMC9311297; doi:10.3389/fmed.2022.916134)

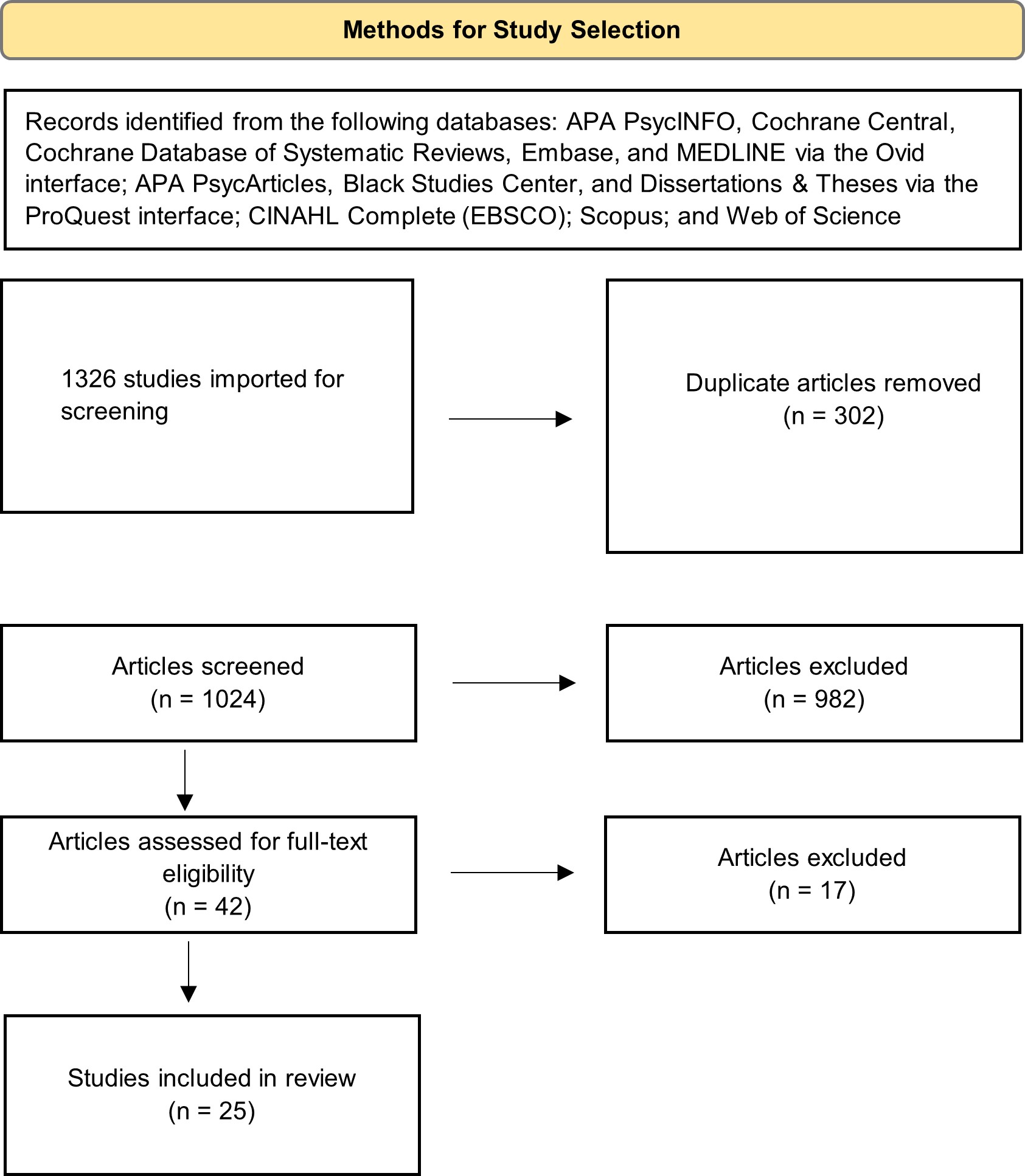

Supplement: Supplementary Figure 1 — Study selection and screening methods used in present study. Figure was adapted from the PRISMA Flow Diagram 2020 V1. Inclusion criteria included studies with cohorts of CLE patients (with or without SLE) whose primary outcome included CLE diagnosis frequency, CLE disease outcomes or health-related quality of life. Exclusion criteria included non- English studies, conference abstracts, and SLE only cohorts. Searches were completed on February 2, 2022. There were no publication date limits. Covidence review platform was used for study screening and extraction. Two separate reviewers (AW and GL) independently appraised all studies meeting inclusion and exclusion criteria. 1,326 studies were imported to Covidence for abstract and title screening. 302 duplicates were removed and then 1,024 studies were left for screening. 982 articles were excluded based on exclusion criteria. 42 articles were assessed for full-text eligibility and 17 studies were excluded, leaving 25 studies total included in this review. Information regarding primary research objective was then extracted from all full text articles and summarized in text. [file Image_1.JPEG]
